# *Notes from the Field*: Use of Social Media as a Communication Tool During a Mumps Outbreak — New York City, 2015

**DOI:** 10.15585/mmwr.mm6602a5

**Published:** 2017-01-20

**Authors:** Beth M. Isaac, Jane R. Zucker, Jennifer MacGregor, Mekete Asfaw, Jennifer L. Rakeman, Jie Fu, Bisram Deocharan, Dakai Liu, Jennifer B. Rosen

**Affiliations:** ^1^Bureau of Immunization, New York City Department of Health and Mental Hygiene; ^2^Council of State and Territorial Epidemiologists/CDC Applied Epidemiology Fellowship; ^3^National Center for Immunization and Respiratory Diseases, CDC; ^4^Bureau of Communications, New York City Department of Health and Mental Hygiene; ^5^Public Health Laboratory, New York City Department of Health and Mental Hygiene.

On August 16, 2015, a case of parotitis in a resident of the Rockaways neighborhood of Queens, New York City (NYC), was reported to the NYC Department of Health and Mental Hygiene (DOHMH) as a suspected mumps case. Subsequent investigations by DOHMH discovered an outbreak of mumps in the Rockaways, with 52 confirmed and probable mumps cases. DOHMH conducted a Facebook advertisement campaign providing information about mumps and the outbreak, which was targeted to Facebook users in the Rockaways neighborhood. The advertisement was shown to 86,111 persons during an approximately 2-week period and provided a timely and inexpensive means of effectively communicating with a large, targeted population.

After the case of parotitis was reported on August 16, 2015, DOHMH identified two additional cases through investigation of the patient’s close contacts. These cases were the first indication to DOHMH of a mumps outbreak in the Rockaways. Because the first patient mentioned other persons in the neighborhood with parotitis, DOHMH contacted health care providers in the Rockaways for information about other patients with parotitis and any mumps laboratory testing not previously reported.

DOHMH conducted investigations through interviews and review of medical records. Diagnostic testing included identification of mumps immunoglobulin M (IgM) in serum and detection of mumps virus RNA by real-time reverse–transcription polymerase chain reaction (rRT-PCR) of buccal swabs. DOHMH used criteria from the Council of State and Territorial Epidemiologists to classify cases as confirmed, probable, or discarded (*1*). Cases were identified through routine provider and laboratory reports to DOHMH; six additional cases were identified through retrospective case finding. Although mumps is a nationally notifiable disease, providers had not reported these cases to DOHMH because they did not suspect mumps or because IgM testing was negative and rRT-PCR testing was not done.

Overall, the outbreak included 52 confirmed and probable mumps cases, with illness onset from June 19–November 2, 2015. Forty-seven patients lived in the Rockaways, and five lived elsewhere in NYC. Two patients who lived outside NYC were not included in this analysis. Median age of mumps patients was 31 years (range = 4–69 years); all but two were adults aged ≥18 years. No patients were hospitalized or had complications. Among the 50 cases for which laboratory testing was conducted, 32 (64%) tested positive by any test, including 29 (66%) of 44 tested by rRT-PCR, and seven (15%) of 47 tested for IgM. Twenty-five (48%) patients had evidence of prior immunity (2 documented doses of mumps-containing vaccine, positive immunoglobulin G titers, or birth before 1957), and immune status of 27 (52%) patients was unknown. Twenty-two patients reported having attended several common neighborhood bars and restaurants during either their incubation or infectious period. Initial control measures implemented during August–November, 2015, included home isolation of infectious patients, notifications to health care providers in the Rockaways, provision of vaccine to two local clinics for administration at no cost, and distribution of informational posters and flyers throughout the neighborhood, specifically targeting the common bars and restaurants attended by patients.

Because DOHMH continued to receive mumps reports from the Rockaways during October despite usual control measures, DOHMH conducted a Facebook advertisement campaign targeted to Facebook users aged 20–59 years in the Rockaways zip codes, as determined by accounts’ home addresses, Internet Protocol addresses used to access the Internet, or locations of mobile devices. The advertisement provided information about mumps and the outbreak and instructed persons with symptoms to stay home, and was shown to 86,118 unique persons during its run (October 30–November 17, 2015). It was clicked on 4,085 times and received 954 likes, 297 comments, and 843 shares, which was more shares than any other DOHMH post at that time. The $3,200 cost was less than that for traditional print media, and the advertisement could be placed quickly and removed once the outbreak had concluded. DOHMH responded to commenter questions in real time, necessitating the availability of DOHMH personnel to respond quickly. Social media provided a timely and inexpensive means for successfully and rapidly communicating with a large population in the target demographic and facilitating public engagement with DOHMH about the mumps outbreak, and therefore, might be useful for disseminating messages to a targeted population during future outbreaks.
